# Social Media Engagement and HIV Testing Among Men Who Have Sex With Men in China: A Nationwide Cross-Sectional Survey

**DOI:** 10.2196/jmir.7251

**Published:** 2017-07-19

**Authors:** Bolin Cao, Chuncheng Liu, Maya Durvasula, Weiming Tang, Stephen Pan, Adam J Saffer, Chongyi Wei, Joseph D Tucker

**Affiliations:** ^1^ School of Media and Communication Shenzhen University Shenzhen China; ^2^ University of North Carolina Project - China Guangzhou China; ^3^ Sanford School of Public Policy Duke University Durham, NC United States; ^4^ University of North Carolina at Chapel Hill Chapel Hill, NC United States; ^5^ School of Media and Journalism University of North Carolina at Chapel Hill Chapel Hill, NC United States; ^6^ University of California, San Francisco San Francisco, CA United States

**Keywords:** social Media, HIV, China, homosexuality, male, mobile application

## Abstract

**Background:**

Many interventions find that social media engagement with health promotion materials can translate into behavioral changes. However, only a few studies have examined the ways in which specific actions on various social media platforms are correlated with health behaviors.

**Objective:**

The objective of this study was to examine the association between social media use and HIV testing behaviors among Chinese men who have sex with men (MSM).

**Methods:**

In July 2016, a Web-based survey was conducted to recruit MSM in 8 Chinese cities through Blued (Blue City Holdings Ltd.), the world’s largest gay mobile phone app. Data on sociodemographic variables, social media use platforms and behaviors, sexual behaviors, and HIV testing histories were collected. HIV testing–related social media use was defined as having ever engaged with HIV testing content on social media, which was further divided into observing (ie, receiving), endorsing (eg, liking and sharing), and contributing (eg, posting or commenting on HIV testing materials). Confirmatory factor analysis (CFA) was conducted to determine the best division of HIV testing–related social media use. Univariate and multivariable logistic regressions were used to examine the association between HIV testing–related social media use and HIV testing behaviors.

**Results:**

A total of 2105 individuals participated in the survey. Among them, 46.75% (984) were under the age of 24 years, 35.43% (746) had high school education or less, and 47.74% (587) had condomless sex in the last 3 months. More than half of the respondents (58.14%, 1224/2105) reported HIV testing–related social media use. Additionally, HIV testing–related social media use, especially on multifunctional platforms such as WeChat, was found to be associated with recent HIV testing (adjusted odds ratio [aOR] 2.32, 95% CI 1.66-3.24). Contributing on social media was correlated with recent HIV testing (aOR 2.10, 95% CI 1.40-3.16), but neither observing (aOR 0.66, 95% CI 0.38-1.15) nor endorsing (aOR 1.29, 95% CI 0.88-1.90) were correlated.

**Conclusions:**

Our data suggest that social media use, particularly on multifunctional platforms such as WeChat and with contributing behaviors, is correlated with HIV testing among MSM in China. Campaigns that promote active participant contribution on social media beyond passive observation and endorsement of promotional materials are needed. This study has implications for the design and implementation of social media interventions to promote HIV testing.

## Introduction

Social media components are increasingly being integrated into public health interventions. Web-based engagement with social media health promotion campaigns can translate into offline behavioral changes [1,2]. Mobile phone apps and related social media provide opportunities for men to locate potential sex partners in their vicinity; according to recent statistical data, men report opening such apps approximately 190 times per week [3]. Healthcare professionals are increasingly using social media to develop health promotion materials, distribute information, and establish peer-mentored education programs [4]. A wide range of social media interventions have been successfully implemented to improve health worldwide [5-7].

Although the effectiveness of social media interventions is well established, the relationship between social media engagement and relevant behavioral outcomes has not been fully explored [8,9]. In particular, detailed examination of the ways in which participants engage with social media platforms in public health interventions is scarce [10-12]. Functions and features vary between different social media platforms, with different platforms encouraging specific types of engagement (sharing, liking, commenting, etc). Identifying the effects of specific social media platforms and behaviors on health outcomes and taking advantage of them can help optimize health-related intervention effects [13].

For HIV prevention, low HIV testing rate is a major obstacle and a disruption to the continuum of care [14,15]. A large subgroup of men who have sex with men (MSM) and engage in high-risk sexual behaviors has never tested for HIV [16], thereby resulting in a sizable population of HIV-positive men who do not know their status [17,18]. Studies on MSM in the United States, the United Kingdom, and Thailand using social and sexual networking sites have demonstrated that social media interventions for HIV testing promotion are both feasible and effective [19-21]. Social media platforms, which are used by MSM across the world to expand social circles, help build community, find sexual partners [22,23], and allow public health practitioners to implement HIV testing promotion among MSM [12,21,24].

In China, the rise in popularity of Web-based social and sexual networking sites has coincided with an increase in the prevalence of HIV infection among MSM in recent decades [25]. Social media platforms such as Weibo, WeChat, QQ, and gay apps are providing men with social and sexual networking opportunities and mediums for learning new information. In 2015, WeChat, a multifunctional social media platform based in China, reported 549 million monthly active users [26]. Meanwhile, between 2006 and 2014, the HIV infection rate among MSM rose from 2.5% to 25.8% [27]. As social media platforms are increasingly being integrated into Chinese daily life and public health interventions, there is a clear need for greater analysis of the specific effects of various forms of social media use on offline HIV testing behaviors [27,28]. This study uses data from a cross-sectional Web-based survey to examine the association between social media use by Chinese MSM and HIV testing behaviors.

## Methods

### Recruitment

We conducted a Web-based survey among MSM in 8 Chinese cities: Guangzhou, Shenzhen, Zhuhai, and Jiangmen (Guangdong Province, Southern China); and Jinan, Qingdao, Yantai, and Jining (Shandong Province, Northern China) in July 2016. This was a baseline survey of an intervention study to evaluate the promotion of HIV testing among MSM in China, and these 8 cities were chosen because they were urban cities with relatively high rates of HIV prevalence [27]. Protocol of the study was registered in the Clinical Trials.gov database (NCT02796963). We followed the Checklist for Reporting Results of Internet E-Surveys for reporting the development and findings of Web-based surveys [29].The pretest survey was field tested by 30 MSM in April 2016, and their feedback was taken into account to finalize the survey questionnaire.

Participants were recruited through Blued, the world’s largest gay mobile app [30]. Private short messages containing the survey link were sent to the registered Blued users in the 8 cities. The study used cellphone numbers to prevent multiple entries from the same individual. Duplicated responses were excluded from the analysis. The eligible participants included those who were born biologically male, had ever engaged in anal sex with a man, were at least 16 years of age, were currently living in one of the designated 8 cities, were willing to provide their cell phone number, and were willing to complete an informed consent procedure. All eligible participants signed an electronic informed consent form before completing the survey and received a small phone card reimbursement or WeChat Red Envelope (hongbao), equivalent to roughly US $7.5.

### Measures

Sociodemographic information collected in the survey (full survey attached as Multimedia Appendix 1) included age, education, income, and marital status. Questions on sexual orientation and disclosure of sexual orientation to others were also included. Participants were asked about their sexual histories and behaviors, including the number of sex partners and condom use practices in the past 3 months. The survey also included questions on HIV testing behaviors, including past HIV testing (beforethe recent 3 months) and testing for HIV in the preceding 3 months. We focused on HIV testing in the past 3 months as outcome to reduce recall bias and to remain consistent with questions about social media use within the past 3 months. These measures had been used in previous Web-based surveys among MSM in China [31].

The survey included a section on social media use, in which participants were asked whether they had ever engaged with any content related to HIV testing on social media in the past and, specifically, whether they had done so in the past 3 months. In this study, we defined HIV testing–related social media use as having ever engaged on a social media platform with any information or materials that mention or promote HIV testing.

Men who reported HIV testing–related social media use were further asked about specific behaviors on 4 social and sexual networking platforms: Weibo, QQ, WeChat, and gay-specific dating apps. Weibo is a Chinese microblogging platform, similar to Twitter, where users can publicly broadcast short messages (under 140 Chinese characters) to their friends and followers [32]. Both QQ and WeChat are Chinese instant messaging platforms that provide multiple functions, including communication, information, entertainment, and financial services [33]. Whereas QQ is optimized for desktop use [34], WeChat is optimized for mobile phone use [26]. Gay-specific dating and networking apps mainly refer to Blued, a Chinese app with 27 million registered users. Other gay apps included Hornet, Grindr, and Zank, which are all available on mobile phones and allow participants to meet new sexual partners [35].

In addition, men who reported HIV testing–related social media use in the past 3 months were asked whether they had ever received, liked, or commented on information related to HIV testing or whether they had ever shared materials on their own timelines, forwarded them to others, or discussed them in one-on-one or group messages on various social and sexual networking platforms. These categories of social media activities were adopted and revised from previous studies, which distinguish behaviors based on the differing levels of time investment and effort required [36,37]. In particular, specific social media use behaviors in the past 3 months were categorized into three groups: observing, which included receiving HIV testing information; endorsing, which included liking HIV testing materials, forwarding them to others, and sharing them on timelines; and contributing, which included posting original information, commenting on someone else’s post about HIV testing, and participating in one-on-one or group chats [38,39].

### Statistical Analysis

Descriptive statistics were used to describe men’s sociodemographic information, sexual risk behaviors, and social media use. Bivariate analysis was used to examine factors associated with HIV testing–related social media use.

Confirmatory factor analysis (CFA) was conducted to confirm the division of social media use into more specific categories. The indicators of CFA included the chi-squared test of minimum discrepancy divided by degrees of freedom (CMIN/DF), goodness of fit index (GFI), adjusted goodness of fit index (AGFI), normed fit index (NFI), comparative fit index (CFI), and root mean square error of approximation (RMSEA). The CMIN/DF assesses the fit of a model in CFA and modeling in which the minimum discrepancies are divided by its degrees of freedom. The value of CMIN/DF should ideally be less than 2.0. The GFI is a measure of fit between the hypothesized model and the observed covariance matrix; the AGFI corrects GFI, which is affected by the number of indicators of each latent variable. The GFI and AGFI range between 0 and 1, with a value of over .9 generally indicating an acceptable model fit. The NFI analyzes the discrepancy between the chi-squared value of the hypothesized model and the chi-squared value of the null model. Values for the NFI should range between 0 and 1, with a cutoff of .95 or greater indicating a good model fit. The CFI analyzes the model fit by examining the discrepancy between the data and the hypothesized model while adjusting for the issues of sample size. The CFI values range from 0 to 1, with larger values indicating a better fit. A CFI value of .95 or higher is presently accepted as an indicator of good fit. The RMSEA examines the discrepancy between the hypothesized model with optimally chosen parameter estimates and the population covariance matrix. The RMSEA ranges from 0 to 1, with smaller values indicating a better model fit. A value of .06 or less is indicative of an acceptable model fit.

Logistic regressions (univariate and multivariable) were used to examine the relationship between HIV testing–related social media use and HIV testing behaviors. Multivariable logistic regression models were adjusted for potential confounding variables, including age, education, income, marital status, sexual orientation, disclosure of sexual orientation to others, partner status (primary partner, multiple partners, etc) and past HIV testing (before the recent 3 months). As social media platforms are designed for and encourage different actions and behaviors, this study controlled for platforms while examining the effect of specific Web-based actions on offline behavior [40,41].

### Ethical Statement

This study was approved by the ethics review committees at the Guangdong Provincial Center for Skin Diseases and STI Control; the University of North Carolina, Chapel Hill (14-1685); and the University of California, San Francisco (14-14877) before the survey launch.

## Results

### Study Participants

The survey link distributed on Blued was clicked 36,863 times in 3 days, with 25,141 unique Internet Protocol addresses automatically collected by the survey tool. Of these clicks, 2112 individuals met the study eligibility criteria and completed the questionnaire in its entirety. Seven responses were deleted as duplicates. Overall, 2105 eligible men from the 8 designated cities were included in the final analysis. The percentages of participants who were from Guangzhou, Shenzhen, Zhuhai, Jiangmen, Jinan, Qingtao, Yantai, and Jining were 14.68% (309), 14.87% (313), 11.21% (236), 9.07% (191), 13.21% (278), 13.92% (293), 12.92% (272), and 10.12% (213), respectively.

### Demographics and Behaviors

Of the 2105 men who participated in the survey, 46.75% (984) were under the age of 24 years, and 35.44% (746) had high school education or less. Most men (85.94%, 1809) were never married, and 61.24% of men (1289) had an annual income over US $5500.

**Table 1:** Sociodemographic and behavioral characteristics of men who have sex with men in 8 Chinese cities, 2016 (N=2105).

Characteristics		Total, n (%)
**Age (years)**		
	≤24	984 (46.75%)
	>24	1121 (53.25%)
**Education**		
	High school or below	746 (35.44%)
	Some college	583 (27.70%)
	College or above	776 (36.86%)
**Annual income (US $)**		
	<2700	391 (18.57%)
	2700-5500	425 (20.19%)
	5501-9200	690 (32.78%)
	9201-15,000	384 (18.24%)
	>15,000	215 (10.21%)
**Marital status**		
	Never married	1809 (85.94%)
	Ever married	296 (14.06%)
**Sexual orientation**		
	Gay	1524 (72.40%)
	Bisexual	496 (23.56%)
	Heterosexual	11 (0.53%)
	Unsure	74 (3.52%)
**Ever disclosed sexuality to others**		
	Yes	1426 (67.74%)
	No	679 (32.26%)
**Main partner in the past 3 months**		
	Yes	812 (38.57%)
	No	1293 (61.43%)
**Number of sexual partners in the past 3 months**		
	0-1	1477 (70.17%)
	Multiple	628 (29.83 %)
**Consistent condom use in the past 3 months^a^**		
	Yes	643 (52.28%)
	No	587 (47.72%)
**Past HIV testing before the recent 3 months**		
	Yes	628 (29.83%)
	No	1477 (70.17%)
**Tested for HIV in the past 3 months**		
	Yes	687 (32.64%)
	No	1418 (67.36%)
**Ever HIV testing–related social media use**		
	Yes	1224 (58.15%)
	No	881 (41.85%)
**HIV testing–related social media use in the past 3 months**		
	Yes	954 (45.32%)
	No	1151 (54.68%)

^a^Men who reported having sex within the past 3 months were asked about consistent condom use (N=1230).

Nearly three-fourths (72.40%, 1524) of men self-identified as gay, and over two-thirds (67.74%, 1426) had ever disclosed their sexual orientation to others. Over one-third (38.57%, 812) reported a main sexual partner in the past 3 months. Nearly one-third (29.83%, 628) had multiple sexual partners, and 47.72% (587) did not consistently use condoms among the men who had sex in the last 3 months. Nearly one-third (29.83%, 628) of respondents had tested for HIV before the recent 3 months, and 32.64% (687) had been tested in the past 3 months. Among respondents, 58.15% (1224) reported ever HIV testing–related social media use, and 45.32% (954) reported such experience in the last 3 months (Table 1).

### Specific HIV Testing–Related Social Media Use Categories

CFA indicated that the model of specific social media use categories had a good fit (CMIN/DF=2.56, GFI=.99, AGFI=.98, NFI=.99, CFI=.99, RMSEA=.04). Of the three social media use categories, observing occurred most frequently on gay mobile apps (460, 48.2%), followed by WeChat (396, 41.5%). Endorsing occurred most frequently on WeChat: 26.8% (256) had liked HIV testing–related information, 17.2% (164) had forwarded such information to others, and 18.6% (177) had shared HIV-related materials on their own timelines. Contributing was a common behavior on both WeChat and QQ. Men posted original information about HIV testing on WeChat (154, 16.1%) and QQ (146, 15.3%); commented on others’ posts on WeChat (186, 19.5%) and QQ (150, 15.7%); had one-on-one chats about HIV testing on WeChat (265, 27.8%) and QQ (237, 24.8%); and had group chats on WeChat (226, 23.7%) and QQ (247, 25.9%). Men tended to be least likely to use Weibo to conduct HIV testing–related behaviors (Table 2).

### Correlates of HIV Testing–Related Social Media Use

In bivariate analyses, age, education, income, and marital status were not correlated with HIV testing–related social media use. Self-identified gay men reported HIV testing–related social media use in the past 3 months more frequently than heterosexual and bisexual men (74.5% vs 70.63%, χ^2^_1_=3.9, *P*=.047). Men who had ever disclosed sexual orientation to others were more likely to report HIV testing–related social media use (73.2% vs 63.25%, χ^2^_1_=23.5, *P*<.001). Men who had main partners in the past 3 months were more likely to report HIV testing–related social media use (41.8% vs 35.88%, χ^2^_1_=7.8, *P*=.01).

Compared with men without HIV testing–related social media use, men with HIV testing–related social media use were more likely to have had multiple sexual partners in the past 3 months (33.2% vs 27.02%, χ^2^_1_=9.6, *P=*.01), and have been recently tested for HIV (40.5% vs 26.15%, χ^2^_1_=48.6, *P*<.001) (Table 3).

**Table 2:** Specific social media use platforms and behaviors in the past 3 months among men who have sex with men in 8 Chinese cities, 2016 (N=954). Weibo is a Chinese microblogging platform, akin to Twitter; QQ and WeChat are Chinese instant messaging platforms, with QQ largely optimized for desktop computers and WeChat optimized for mobile phones; gay apps refer to gay-specific networking apps.

Behaviors		Weibo, n (%)	WeChat, n (%)	QQ, n (%)	Gay apps, n (%)
**Observing**					
	Received information about HIV testing	227 (23.8%)	396 (41.5%)	318 (33.3%)	460 (48.2%)
**Endorsing**					
	Liked information about HIV testing	180 (18.9%)	256 (26.8%)	189 (19.8%)	240 (25.2%)
	Forwarded information about HIV testing to others	120 (12.6%)	164 (17.2%)	139 (14.6%)	141 (14.8%)
	Shared information on timeline about HIV testing	108 (11.3%)	177 (18.6%)	134 (14.0%)	146 (15.3%)
**Contributing**					
	Posted original information about HIV testing	90 (9.4%)	154 (16.1%)	146 (15.3%)	138 (14.5%)
	Commented on others’ post about HIV testing	103 (10.8%)	186 (19.5%)	150 (15.7%)	179 (18.8%)
	One-on-one chatted about HIV testing	101 (10.6%)	265 (27.8%)	237 (24.8%)	206 (21.6%)
	Group chatted about HIV testing	85 (8.9%)	226 (23.7%)	247 (25.9%)	175 (18.3%)

**Table 3:** Comparison of sociodemographic and behavioral characteristics between men with HIV testing–related social media use and men without among men who have sex with men in 8 Chinese cities, 2016 (N=2105).

Sociodemographic and behavioral characteristics			Men with HIV testing– related social media use (N=954), n (%)		Men without HIV testing– related social media use (N=1151), n (%)		Chi-square		*P* value
**Age, in years**									
	≤24	433 (45.4)		551 (47.87)		1.3		.26	
	>24	521 (54.6)		600 (52.13)					
**Education**									
	High school or below	305 (32.0)		441 (38.31)		9.2		.01	
	Some college	278 (29.1)		305 (26.50)					
	College or above	371 (38.9)		405 (35.19)					
**Annual income (US $)**									
	<2700	165 (17.3)		226 (19.64)		5.8		.21	
	2700-5500	195 (20.4)		230 (19.98)					
	5501-9200	313 (32.8)		377 (32.75)					
	9201-15,000	169 (17.7)		215 (18.68)					
	>15,000	112 (11.7)		103 (8.95)					
**Marital status**									
	Never married	813 (85.2)		996 (86.53)		0.8		.21	
	Ever married	141 (14.8)		155 (13.47)					
**Sexual orientation**									
	Gay	711 (74.5)		813 (70.63)		4.0		.047	
	Others^a^	243 (25.5)		338 (29.37)					
**Ever disclosed sexuality to others**									
	Yes	698 (73.2)		728 (63.25)		23.5		<.001	
	No	256 (26.8)		423 (36.75)					
**Main partner in the past 3 months**									
	Yes	399 (41.8)		413 (35.88)		7.8		.01	
	No	555 (58.2)		738 (64.12)					
**Number of sexual partner(s) in the past 3 months**									
	0-1	637(66.8)		840 (72.98)		9.6		.01	
	Multiple	317 (33.2)		311 (27.02)					
**Consistent condom use in the past 3 months**									
	Yes	329 (55.0)		314 (49.68)		3.5		.06	
	No	269 (45.0)		318 (50.32)					
**Past HIV testing before the recent 3 months**									
	Yes	277 (29.0)		351 (30.50)		0.5		.47	
	No	677 (71.0)		800 (69.50)					
**Tested for HIV in the past 3 months**									
	Yes	386 (40.5)		301 (26.15)		48.6		<.001	
	No	568 (59.5)		850 (73.85)					

^a^Others refers to heterosexual and bisexual.

**Table 4:** Correlations between HIV testing–related social media use and HIV-related behaviors among men who have sex with men in 8 Chinese cities, 2016 (N=2105).

HIV-related behaviors		Recent HIV testing–related social media use
		Adjusted odds ratio (95% CI)
**Number of sexual partner(s) in the past 3 months**		
	0-1	Ref
	Multiple	1.23^a^ (1.01-1.50)
**Consistent condom use in the past 3 months**		
	Yes	1.28^a^ (1.01-1.61)
	No	Ref
**Tested for HIV in the past 3 months**		
	Yes	2.02^b^ (1.63-2.52)
	No	Ref

^a^*P*<.05.

^b^*P*<.001.

### Multivariable Analyses of Social Media Use and HIV Testing

Multivariable logistic analysis showed that HIV testing–related social media use was significantly associated with the number of sexual partners (adjusted odds ratio [aOR]1.23, 95% CI1.01-1.50) and consistent condom use behaviors (aOR1.27, 95% CI1.01-1.60) in the past 3 months after adjusting for potential confounders. Furthermore, HIV testing–related social media use was also significantly associated with recent HIV testing (aOR2.02, 95% CI 1.63-2.52; Table 4).

Multivariable analysis controlled for age, education, income, marital status, sexual orientation, disclosure status, main partner in the past 3 months, and past HIV testing before the recent 3 months.

There was a platform-linked variation in the relationship between HIV testing–related social media use and HIV testing behaviors in the past 3 months (Table 5). WeChat use was significantly associated with recent HIV testing (aOR2.32, 95% CI 1.66-3.24), but Weibo (aOR 0.88, 95% CI 0.62-1.25), QQ (aOR1.34, 95% CI 0.96-1.86), and gay app (aOR0.80, 95% CI 0.57-1.11) use were not. Table 5 also indicated the relationship between specific HIV testing–related social media use and recent HIV testing behaviors. Contributing on social media was significantly associated with recent HIV testing (aOR2.10, 95% CI 1.40-3.16). Endorsing on social media was significantly correlated with recent HIV testing in bivariate analysis (Crude OR1.46, 95% CI 1.11-1.92) but not in multivariable analysis (aOR1.29, 95% CI0.88-1.90).

Multivariable analysis controlled for age, education, income, marital status, sexual orientation, disclosure status, main partner in the past 3 months, and past HIV testing.

**Table 5:** Correlations between specific social media use platforms and HIV testing behaviors in the past 3 months among men who have sex with men in 8 Chinese cities, 2016 (N=954).

Characteristic			Recent HIV testing					
			n (%)		Crude odds ratio (95% CI)		Adjusted odds ratio (95% CI)	
**Social media use platforms**								
	**Weibo use**							
		Yes		307 (32.2%)		0.98 (0.74-1.29)		0.88 (0.62-1.25)
		No		647 (67.8%)				
	**WeChat use**							
		Yes		561 (58.8%)		1.94^a^ (1.48-2.54)		2.32^a^ (1.66-3.24)
		No		393 (41.2%)				
	**QQ use**							
		Yes		464 (48.6%)		1.33^b^ (1.03-1.72)		1.34 (0.96-1.86)
		No		490 (51.4%)				
	**Gay app use**							
		Yes		561 (58.8%)		0.87 (0.67-1.13)		0.80 (0.57-1.11)
		No		393 (41.2%)				
**Social media use behaviors**								
	**Observing^c^**							
		Yes		842 (88.3%)		1.37 (0.91-2.08)		0.66 (0.38-1.15)
		No		112 (11.7%)				
	**Endorsing^c^**							
		Yes		614 (64.4%)		1.46^d^ (1.11-1.92)		1.29 (0.88-1.90)
		No		340 (35.6%)				
	**Contributing^c^**							
		Yes		664 (69.6%)		2.09^a^ (1.56-2.82)		2.10^a^ (1.40-3.16)
		No		290 (30.4%)				

^a^*P*<.001.

^b^*P*<.05.

^c^Multivariable analysis controlled social media platforms that included Weibo, WeChat, QQ, and gay apps.

^d^*P*<.01.

## Discussion

### Principal Findings

The rise of social media has created a set of promising tools for HIV prevention [2,12,21].This study examines the relationship between specific social media engagement and HIV testing behavior, providing information necessary to optimize public health efforts on social media. In particular, this study expands the literature by examining associations between social media use and HIV testing, dividing social media use into specific platforms and behaviors, and examining MSM in a middle-income country. Although several previous studies have treated social media as a generic platform [6,42], this study acknowledges the important versatility of social media platforms and specifically focuses on the ways in which social media use is related to HIV testing.

We found that over half of MSM reported HIV testing–related social media use. This is consistent with past studies that have found that MSM commonly use social media to obtain information about HIV testing [23,42-44]. Additionally, ongoing public health interventions use social media to provide information about HIV testing sites, counseling, and self-testing kits [7,45,46]. As health interventions increasingly seek to integrate technology into healthcare services, there would be more chances for MSM to get engaged with HIV testing on social media [47].

HIV testing–related social media use was positively associated with recent HIV testing. This finding supports the positive association between Web-based engagement with offline health behaviors [48-50]. It is consistent with the literature from other fields (eg, smoking cessation) showing that social media use is related to offline behaviors [51-53]. Social media has several key advantages, which encourage this link between Web-based engagement and offline behavior. In particular, social media platforms allow public health campaigns to reach more diverse audiences, reduce overall cost, provide opportunities for repeated exposure to messaging, collect real-time feedback, and encourage direct engagement with messaging materials [13]. Men who engage in HIV testing–related social media use may receive detailed information about the testing location, hours of operation, and available services, thus potentially encouraging HIV testing [54].

Certain social media platforms and behaviors are strongly linked to recent HIV testing. WeChat use, in particular, was strongly correlated with recent HIV testing in this study. This is consistent with research showing that instant messaging platforms promote HIV testing in China [55] and the United States [56]. In addition, WeChat’s popularity in China and its functional design are particularly conducive to the modes of engagement necessary for impactful public health interventions. On average, an adult in China spends more than 40 min per day on WeChat, and more than half of all users open WeChat more than 10 times per day [57]. The high-frequency use and constant engagement creates many opportunities for target audiences to be exposed to the messaging. Additionally, WeChat’s design, which is optimized for mobile phone use and includes services for information, entertainment, and finance within a single app, encourages users to merge their online and offline activities [26,58]. Some community-based organizations have taken advantage of WeChat’s interactive functions and now allow HIV test appointment scheduling through the app [59]. An ongoing randomized controlled trial is examining the effectiveness of using WeChat to promote HIV testing [60].

Contributing on social media was strongly correlated with recent HIV testing as compared with observing and endorsing. Among the three specific behaviors, observing represents a type of passive involvement that allows men to encounter information without directly engaging [61]. Although receiving information about HIV testing on social media is commonly reported, previous studies have not found this to be associated with HIV testing [62]. Endorsing represents greater engagement, as individuals must consciously present a position of approval or support to certain persons, information, or actions. However, endorsing can include what is often referred to as “slacktivism,” a term indicating actions that are performed online to demonstrate public concern for an issue without requiring significant time or involvement [63]. These public displays of pseudo-engagement may not be conducive to behavioral change, and in fact, may represent detachment from actual behavior by making participants believe that they have already made some significant effort [64]. Compared with observing and endorsing, contributing on social media requires a higher level of cognitive engagement, as individuals need to develop some original perspectives or responses to the issue [65]. Contributing on social media may lead to positive health-related outcomes and influence behavior change [50]. When contributing on social media, men must think through the challenge and cognitively respond to the situation. This contribution process focuses participants’ attention and increases overall comprehension of HIV testing issues, encouraging the translation of Web-based activity to actual behavior [66]. This finding is consistent with the theory that social, participatory, and interactive aspects of social media can promote healthy behavior change [11,67].

This study is particularly relevant for policymakers and researchers who seek to use social media to promote behavior change. Although additional randomized controlled studies are needed to further examine the relationship between social media engagement and offline behavior, this study supports the integration of multifunctional platforms, such as WeChat, into public health interventions. Additionally, researchers and policymakers should encourage authentic input from MSM when designing social media interventions. The active engagement of MSM on social media is preferable to top-down, one-sided health communication [68]. Interactive activities, such as community crowdsourcing contests [69] that encourage higher levels of participation, can be organized in part through social media [70].

### Limitations

This study also has limitations. First, men were recruited from a gay-specific social media platform, likely resulting in overestimation of the rate of social media use. Second, it is a cross-sectional survey, so causal relationships were not established. Response rates and completion rate were not calculated for this Web-based survey. Third, only 8 cities in China were selected; although cities of various sizes were included, the results are not necessarily applicable for rural areas. Given the variation in HIV infection and HIV testing rates between urban and rural areas of China [71], as well as the varying usage of social media as sources of health information [72], future studies are needed to test the feasibility of social media intervention in rural places. Finally, whereas social media platforms share similar characteristics, the landscape of social media tools and functions varies across nations. Further evaluation is needed to understand the feasibility and effectiveness of specific social media use in international contexts.

### Conclusions

Social media interventions have been increasingly incorporated into public health programs [4,12]. This study suggests that Web-based engagement with HIV testing content may spur offline HIV testing behaviors, supporting the role of social media in public health campaigns. Our research suggests that future social media interventions can consider moving beyond merely using social media as a way to disseminate information and should instead leverage the interactive capabilities of various platforms to encourage participation.

## Appendix

Multimedia Appendix 1 Full survey questionnaire.

## Abbreviations

AGFI: adjusted goodness of fit index
aOR: adjusted odds ratio
CFI: comparative fit index
CFA: confirmatory factor analysis
CMIN/DF: chi-squared test of minimum discrepancy divided by degrees of freedom
GFI: goodness of fit index
MSM: men who have sex with men
NFI: normed fit index
RMSEA: root mean square error of approximation
